# Laparoscopic surgery for T4 colon cancer: a systematic review and meta-analysis

**DOI:** 10.1007/s00464-017-5544-7

**Published:** 2017-04-21

**Authors:** Charlotte E. L. Klaver, Tijmen M. Kappen, Wernard A. A. Borstlap, Willem A. Bemelman, Pieter J. Tanis

**Affiliations:** Department of Surgery, Academic Medical Centre, University of Amsterdam, Room G4140, Meibergdreef 9, 1105AZ Amsterdam, The Netherlands

**Keywords:** Minimally invasive surgery, Locally advanced colon cancer

## Abstract

**Background:**

In colon cancer, T4 stage is still assumed to be a relative contraindication for laparoscopic surgery considering the oncological safety. The aim of this systematic review with meta-analysis was to evaluate short- and long-term oncological outcomes after laparoscopic surgery for T4 colon cancer, and to compare these with open surgery.

**Methods:**

Using systematic review of literature, studies reporting on radicality of resection, disease-free survival (DFS), and/or overall survival (OS) after laparoscopic surgery for T4 colon cancer were identified, with or without a control group of open surgery. Pooled proportions and risk ratios were calculated using an inverse variance method.

**Results:**

Thirteen observational cohort studies published between 2012 and 2017 were included, together consisting of 1217 patients that received laparoscopic surgery and 1357 with an open procedure. The proportion of multivisceral resections was larger in the open group in five studies. Based on 11 studies, the pooled proportion of R0 resection was 0.96 (95% CI: 0.91–0.99) and 0.96 (95% CI: 0.90–0.98) after laparoscopic and open surgery, respectively. Analysing (mainly) T4a subgroups in 6 evaluable studies revealed pooled R0 resection rates of 0.94 in both groups. No significant differences were found between laparoscopic and open surgery for any survival measure: RR 1.07 (95% CI: 0.96–1.20) for 3-year DFS, RR 1.04 (95% CI: 0.95–1.15) for 5-year DFS, RR 1.07 (95% CI: 0.99–1.14) for 3-year OS, and RR 1.05 (95% CI: 0.98–1.12) for 5-year OS.

**Conclusion:**

Literature on laparoscopic surgery for T4 colon cancer is restricted to non-randomized comparisons with substantial allocation bias. Laparoscopic surgery for T4a tumours might be safe, whereas for T4b colon cancer requiring multivisceral resection it should be applied with caution.

**Electronic supplementary material:**

The online version of this article (doi:10.1007/s00464-017-5544-7) contains supplementary material, which is available to authorized users.

Colon cancer is world-wide a highly prevalent disease [1] and approximately 15% of patients presents with a locally advanced tumour (T4 stage). Resection of T4 colon cancer can be challenging if it directly invades other organs or structures (T4b according to TNM7). This requires a multivisceral resection (MVR) that is conventionally performed using an open approach, but reports on laparoscopic MVRs are increasing.

Laparoscopic surgery for colon cancer was introduced in the late nineties. Several randomized trials showed the short-term benefits of laparoscopic colectomy as compared to open surgery [2–5]. A recent systematic review reported also long-term benefits, with lower risks of adhesion-related small bowel obstruction and incisional hernia [6]. In the multicentre randomized COLOR trial [7], the non-inferiority of laparoscopic surgery for colon cancer in terms of 3-year disease-free survival (3y DFS) was suggested. However, clinically suspected tumour invasion of adjacent structures (cT4b stage) was an exclusion criterion in the COLOR trial. With a reported conversion rate of up to 50% for the remaining T4 tumours, they proposed that an open approach could be more appropriate for T4 colon cancer.

Laparoscopic colectomy for colon cancer has become widely accepted and implemented in routine practice, but T4 stage still constitutes a relative contraindication for laparoscopic surgery. A laparoscopic approach might jeopardize radicality of the resection, with its impact on long-term oncological outcome [8]. The aim of this study was to systematically review the currently available literature on laparoscopic surgery for T4 colon cancer, with regard to short- and long-term oncological outcomes, and to compare these with open surgery.

## Methods

### Literature search

A systematic search was performed on the 21st of February 2017. Studies describing laparoscopic surgery for T4 colon cancer were identified using MEDLINE, EMBASE, and PubMed using the search terms as provided in Supplementary Table 1. The review protocol was developed according to the PRISMA (Preferred Reporting Items for Systematic Reviews and Meta-Analyses) guidelines [9]. Two researchers independently screened titles and abstracts to select articles for full-text reading. In case of disagreement, discussion took place until consensus was reached. Relevant review articles and bibliographies of included studies were reviewed for additional relevant publications. No restrictions with respect to publication date were applied. Subsequently, two researchers performed full-text reading for the definitive selection of articles.

### Study selection and quality assessment

Articles were considered eligible if containing original data on laparoscopic resection of T4 colon cancer. T4 stage referred to either clinical or pathological stage. If studies included both colon and rectal cancer without separate data on outcome measures, authors were contacted to provide data for the relevant subgroup. If these were not available, studies were excluded. Furthermore, all authors were contacted to provide separate outcome data for the pT4a and pT4b subgroups.

Radicality of resection, postoperative morbidity and long-term oncological survival outcomes were considered the main study outcomes. Studies that did not report on at least one of the main outcome measures were excluded. Both comparative (laparoscopic versus open surgery) and non-comparative studies were eligible. Case reports and studies with less than 10 patients were excluded. The quality of the included articles was independently assessed by two authors using the nine-point Newcastle–Ottawa Scale [10].

### Data extraction

General study characteristics (i.e. study design, in/exclusion criteria), baseline patient and tumour characteristics, operative characteristics (setting, need for conversion, MVR), and data on multimodality treatment (i.e. systemic therapy) were extracted from the included studies. Outcome measures included postoperative complications (<30 days, not further predefined), postoperative mortality, radicality of resection, disease-free survival (DFS), and overall survival (OS). In case of comparative studies, relevant data were also extracted for the subgroup that received open surgery for T4 colon cancer. If available, data on oncological outcomes were retrieved for T4a and T4b stages separately. Data extraction was performed by two researchers independently.

### Statistical analyses

Pooled averages were calculated for perioperative outcome measures. Pooled proportions with 95% confidence intervals (95%CI) were calculated using an inverse variance method (random effects model) in RStudio version 3.2.3 (R Foundation for Statistical Computing, Vienna, Austria 2016). For the purpose of comparing laparoscopic with open surgery, risk ratios were calculated with corresponding 95% confidence intervals (CI) using an inverse variance method (random effects model). The *I* ^2^ statistic was calculated in order to evaluate heterogeneity of the included studies. Meta-analyses were performed using Review Manager 5.3 (The Nordic Cochrane Centre, The Cochrane Collaboration, Copenhagen, Denmark 2014). A *p* value of less than 0.05 was considered statistically significant.

## Results

### Included studies

A total of 2689 hits were identified by searching MEDLINE, EMBASE, and Pubmed. The further selection process is displayed in Supplementary Fig. 1 and led to 56 articles eligible for full-text reading. Finally, 13 studies (23%) were included, all published between 2012 and 2017 (Table 1) [11–23]. There were no randomized controlled trials. Twelve studies were comparative observational cohort studies, including three studies applying matching or weighting. De’Angelis [11] used propensity score matching including the following variables in the regression model: age, sex, ASA score, surgical procedure, tumour location, tumoural and nodal stage, tumour size, MVR, and year of surgery. Elnahas [13] used inverse probability of treatment weighting (IPTW) creating a propensity score that represents the probability a patient was approached with laparoscopic resection, including age, sex, BMI, high ASA (>2), renal failure, chronic obstructive pulmonary disease (COPD), congestive heart failure (CHF), bleeding disorder, preoperative chemotherapy, metastasis, nodal disease, rectosigmoid resection, and emergency cases. Vignali [22] performed a case-matched control study and matched for disease stage (II/III/IV), ASA score, year of surgery (±3 years), and sex.

**Table 1:** Characteristics of the included studies

References	Design	Population	Exclusion criteria	Sample size
De’Angelis et al. [11]	Retrospective, two centers, comparative, PSM, ITT	pT4 colon cancer (TNM7)	Emergency procedures M1	Lap: 106 Conv: 13 (12%) Open: 106
Chan et al. [12]	Retrospective, single center, comparative, ITT	pT4 colon cancer (TNM6/7)	Emergency proceduresM1 Direct invasion to adjacent organs	Lap: 93 Conv: 8 (8.6%) Open: 59
Elnahas et al. [13]	Retrospective, multicenter, comparative, IPTW, ITT	pT4a colon cancer (TNM7)	T4b; Tumor below peritoneal reflection	Lap: 455 Conv: 8 (11%) Open: 406
Kang et al. [14]	Retrospective, single center, comparative, ITT	pT4 colon cancer (TNM7)	M1 Robotic surgery	Lap: 52 Conv: 4 (7.7%) Open: 57
Kim et al. [15]	Retrospective, single center, comparative, ITT	pT4 colon cancer (TNM7)	Non-resectional surgery; Bypass surgery; FAP; HNPCC M1	Lap: 51 Conv: 7 (14%) Open: 66
Nagasue et al. [16]	Prospective, single center, comparative, ITT	Patients receiving MVR for colorectal cancer (TNM7), *Separate data on colon only included in this analysis*	Emergency surgery; Synchronous resection of liver metastases; Pelvic exenteration; Pancreaticoduodenectomy; Distal pancreatectomy: Resection of spleen, ureter, gallbladder, neurovascular bundle, psoas muscle, or sacrum. Palliative resections;	Lap: 39 Conv: 3 (7.7%) pT4: 30 (77%) Open: 53 pT4: 41 (77%)
Park et al. [17]	Retrospective, single center, comparative, ITT	cT4 colon cancer (TNM7)	Recurrent cancer M1 FAP, HNPCC Neoadjuvant therapy	Lap: 71 Conv: 4 (5.6%) Open: 222
Sammour et al. [18]	Retrospective, multicenter, comparative, ITT	Colon cancer *Separate data on pT4 only included in this analysis*	–	Lap: 89 Conv: 13 (15%) Open: 184
Shukla et al. [19]	Retrospective, single center, comparative, ITT	pT4 colon cancer (TNM7)	M1	Lap: 61 Conv: 13 (21%) Open: 22
Takahashi et al. [20]	Retrospective, single center, ITT	Patients receiving MVR for colon cancer (TNM7)	Recurrent cancer Rectal cancer	Lap: 48 Conv: 6 (13%) Open: 36
Vallribera Valls et al. [21]	Retrospective, single center, comparative, cohort ITT	Colon cancer (TNM6) *Separate data on pT4 only included in this analysis.*	Emergency procedures M1 MVR ASA 4 Previous colonic surgery	Lap: 69 Conv: 4 (5.9%) Open: 76
Vignali et al. [22]	Prospective, single center, comparative, case matched, ITT	pT4 colon cancer (TNM7)	Emergency procedures	Lap: 70 Conv: 5 (7.1%) Open 70
Allaix et al. [23]	Retrospective, single center, non-comparative	Colorectal cancer (TNM7) *Separate data on pT4 colon cancer only included in this analysis*	cM1 Preoperatively evident adjacent organ invasion Obstruction/perforation History of colorectal surgery	Lap: 13 Conv: 3 (23%)

*Conv* conversion; *IPTW* inverse probability of treatment weighting; *ITT* intention to treat analysis; *MVR* multivisceral resection; *PSM* propensity score matching

Three multicentre studies were included and ten single-centre studies. All studies together comprised 1217 patients that received laparoscopic surgery and 1357 that underwent open surgery. In all studies, converted patients were included in the laparoscopic group as intention to treat principle. After contacting the authors of combined colon and rectal cancer series, four studies provided separate data of T4 colon cancer [18, 21, 23, 24]. Regarding pathological versus clinical staging, ten studies included only patients with pT4 stage and inclusion was based on cT4 stage in three studies, with confirmation of pT4 by the pathologist in 77, 38, and 50%. Regarding pathological subgroups of pT4, one study included only pT4a stage [13], and in nine other studies, mainly (>60%) pT4a patients were included [11, 14, 17–19, 21–23, 25]. In two studies, MVR was the main focus, resulting in mostly pT4b stage [16, 20]. In eight studies, patients with metastatic disease were excluded. Emergency procedures were included in five studies [13–15, 18, 19], excluded in six other studies [11, 12, 16, 21–23], and not mentioned in the two remaining studies [17, 20].

Using the Newcastle–Ottawa Scale, two studies scored 9 out of 9 points, one study 8, three studies 7, five studies 6, and two studies were awarded 5 points (Supplementary Table 2).

### Patient, disease, and treatment characteristics

Patient and disease characteristics are displayed in Table 2 for the laparoscopic and open subgroups separately. In Supplementary Table 3, data on operative characteristics are displayed. Conversion rates varied from 5.6 to 23%, with a pooled proportion of 0.11 (95% CI:0.09–0.14). In six of the eight comparative studies that also included MVR [11, 14, 15, 17, 19, 22], the proportion of MVR was higher in the open subgroups and the remaining two comparative studies included 100% MVR [20, 24].

**Table 2:** Patient, disease, and treatment characteristics

References	Group	Age (years)	ASA	T4a/T4b	*N*+-stage	M1-stage	Neoadjuvant therapy	Adjuvant chemotherapy
De’Angelis et al. [11]	Lap (*n* = 106) Open (*n* = 106)	Median: 71 (18–94) Median: 76 (27–91) *p* = 0.208	≥3: 24 (23%) ≥3: 30 (28%) *p* = 0.516	pT4a: 91 (86%) pT4b: 15 (14%) pT4a: 86 (81%) pT4b: 20 (19%) *p* = 0.460	61 (58%) 66 (62%) *p* = 0.573	0 0	NR	63 (59%) 53 (50%) *p* = 0.214
Chan et al. [12]	Lap (*n* = 93) Open (*n* = 59)	NS	NS	NR	54 (58%) 46 (78%) *p* = 0.012	NS	NR	NR
Elnahas et al. [13]	Lap (*n* = 455) Open (*n* = 406)	67 (SD 14) 67 (SD 15) *p* = 0.92	≥3: 27 (6.3%) ≥3: 28 (7.1%) *p* = 0.64	T4a only	296 (69%) 268 (69%) *p* = 0.99	122 (28%) 111 (28%) *p* = 0.98	20 (4.6%) 19 (4.7%) *p* = 0.94	NR
Kang et al. [14]	Lap (*n* = 52) Open (*n* = 57)	62 (SD 14) 65 (SD 12)	≥3: 8 (15%) ≥3: 3 (5.3%)	pT4a: 45 (87%)pT4b: 7 (14%) pT4a: 39 (69%)pT4b: 18 (32%) *p* = 0.025	35 (67%) 33 (58%) *p* = 0.577	0 0	NR	42 (81%) 43 (75%) *p* = 0.502
Kim et al. [15]	Lap (*n* = 51) Open (*n* = 66)	70 (SD 12) 67 (SD 11) *p* = 0.181	≥3: 4 (7.8%) ≥3: 11 (17%) *p* = 0.110	pT4a: 49 (96%) pT4b: 2 (3.9%) pT4a: 54 (82%) pT4b: 12 (18%) *p* = 0.018	34 (67%) 40 (61%) *p* = 0.468	0 0	NR	NR NR
Nagasue et al. [16]	Lap (*n* = 39) Open (*n* = 53)	65 (SD 13) 64 (SD 13) *p* = 0.843	NR	pT1-3: 9 (23%) pT4a: 7 (18%) pT4b: 23 (59%) pT1-3: 12 (23%) pT4a: 4 (7.5%) pT4b: 37 (70%) *p* = 0.275	27 (69%) 21 (39%) *p* = 0.005	15 (39%) 10 (19%) *p* = 0.037	0 (0%) 2 (3.8%) *p* = 0.506	15 (63%) (M0 only (*n* = 24)) 16 (37%) (M0 only (*n* = 43)) *p* = 0.047
Park et al. [17]	Lap (*n* = 71) Open (*n* = 222)	Median: 59 (36–80) Median: 61 (17–84) *p* = 0.321	≥2: 38 (54%) ≥2: 132 (60%) *p* = 0.377	cT4a: 58 (82%) cT4b: 13 (18%) cT4a: 130 (59%) cT4b: 92 (41%) *p* < 0.001	43 (61%) 104 (47%) *p* = 0.085	0 0	0 0	60 (85%) 171 (77%) *p* = 0.179
Sammour et al. [18]	Lap (*n* = 89) Open (*n* = 184)	67 (SD 14) 67 (SD 14) *p* = 0.540	26 (48%)* 53 (44%) *p* = 0.626	pT4a: 48 (81%)*pT4b: 11(19%) pT4a: 80 (67%)pT4b: 40 (33%) *p* = 0.041	66 (74%)* 135 (74%) *p* = 0.997	26 (29%)* 65 (36%) *p* = 0.301	0 (0%) 3 (1.6%) *p* = 0.553	40 (64%) (M0 only (*n* = 63)) 65 (55%) (M0 only (*n* = 118)) *p* = 0.275
Shukla et al. [19]	Lap (*n* = 61) Open (*n* = 22)	Median: 70 (31–88) Median: 71 (35–93) *p* = 0.680	≥3: 27 (44%) ≥3:10 (46%) 0.923	pT4a: 53 (87%) pT4b: 8 (13%) pT4a: 15 (68%) pT4b: 7 (32%) *p* = 0.102	37 (61%) 11 (50%) *p* = 0.667	0 0	1 (1.6%) 2 (9.0%) *p* = 0.170	53 (87%) 16 (73%) *p* = 0.182
Takahashi et al. [20]	Lap (*n* = 48) Open (*n* = 36)	Median: 69 (49–86) Median: 62 (46–88) *p* = 0.135	PS≥1: 11 (23%) PS≥1: 16 (44%) *p* = 0.037	pT4b: 22 (46%) pT4b: 20 (56%) *p* = 0.378	14 (29%) 7 (19%) *p* = 0.397	13 (27%) 8 (22%) *p* = 0.397	(6.3%) (25%) *p* = 0.015	10 (71% of stage III) 4 (57% of stage III)
Vallribera Valls et al. [21]	Lap (*n* = 69) Open (*n* = 76)	71 (SD 12) 70 (SD 12) *p* = 0.612	≥3: 24 (35%) ≥3: 20 (26%) *p* = 0.268		41 (59%) 43 (58%) *p* = 0.873	0 0	NR	NR
Vignali et al. [22]	Lap Open	65 (SD 13) 64 (SD 21) *p* = 0.21	Mean: 1.94 (SD 0.4) Mean: 1.94 (SD 0.6) *p* = 0.88	pT4a: 52 (74%) pT4b: 18 (26%) pT4a: 46 (66%) pT4b: 24 (34%) *p* = 0.49	32 (46%) 32 (46%) *p* = 0.55	5 (7.1%) 5 (7.1%) *p* = 0.55	0 (0%) 0 (0%)	52 (74%) 56 (80%) *p* = 0.66
Allaix et al. [23]	Lap (*n* = 13)	Mean: 60 (7.8)	NR	NR. Mainly T4a, since organ invasion on preoperative imaging was an exclusion criteria	10 (77%)	0	NR	12 (92%)

Age is displayed in median (range) or mean (SD)

*Lap* laparoscopic surgery; *NR* not reported; *Open* open surgery;* PS* performance status

* Excluding missing cases

Postoperative complication rates were described in 11 of the 13 studies, without clear definitions. Postoperative (30-day, early) complication rates ranged from 7.7 to 30% in laparoscopic groups and from 21 to 49% in the open cohorts, mainly including anastomotic leakage, (intra-abdominal) abscess, ileus, urinary tract infection, and wound infection. Pooled proportion of postoperative complications was 0.23 (95% CI: 0.19–0.27) for laparoscopy, and 0.35 (95% CI: 0.31–0.40) for open surgery (Table 4). Pooled postoperative mortality rates were 0.02 (95% CI: 0.01–0.04) and 0.03 (95% CI: 0.02–0.06), respectively. Meta-analysis of comparative studies reporting on postoperative complications revealed a risk ratio for laparoscopic versus open surgery of 0.65 for postoperative complications [95% CI: 0.55–0.77; *p* < 0.001, (heterogeneity: *I* ^2^ 0%, *p* = 0.51)].

Adjuvant chemotherapy was applied in 55–100% of patients after laparoscopic surgery and in 37–80% after open resection. Time to adjuvant chemotherapy was only reported in one study [15]; 34 days (SD9) in the laparoscopic group versus 36 (SD21) days after open resection (*p* = 0.510).

### Oncological outcomes

Eleven studies reported on radicality of resection (R0). Proportions of radical resections varied from 74 to 100% for laparoscopic surgery and from 76 to 100% in the open group (Table 3). Pooled proportions were 0.96 (95% CI: 0.91–0.99) and 0.96 (95% CI: 0.90–0.98), respectively (Table 4). Meta-analysis revealed no significant difference in radicality of resection between laparoscopic and open surgery (RR: 1.00 (95% CI: 0.98–1.01), *p* = 0.80) (Supplementary Fig. 2).

**Table 3:** Oncological outcomes

References	Group	R0 resection rate	Overall survival	Disease-free survival
De’Angelis et al. [11]	Lap (*n* = 106) Open (*n* = 106)	100 (94%) 99 (93%) *p* = 1	3y OS: 77% 5y OS: 59% 3y OS: 70% 5y OS: 60% *p* = 0.864	3y DFS: 66% 5y DFS: 58% 3y DFS: 55% 5y DFS: 50% *p* = 0.261
Chan et al. [12]	Lap (*n* = 93) Open (*n* = 59)	92 (99%) 59 (100%)	3y OS: 70% 3y OS: 54% *p* = 0.335	3y DFS: 59% 3y DFS: 46% *p* = 0.113
Elnahas et al. [13]	Lap (*n* = 455) Open (*n* = 406)	341 (74%) 313 (76%) *p* = 0.24	NR	NR
Kang et al. [14]	Lap (*n* = 52) Open (*n* = 57)	51 (98%) 55 (96%) NS	5y OS: 61% 5y OS: 62% *p* = 0.817	5y DFS: 54% 5y DFS: 63% *p* = 0.980
Kim et al. [15]	Lap (*n* = 51) Open (*n* = 66)	49 (96%) 63 (96%) *p* = 0.869	3y OS: 83% 3y OS: 76% NS	3y DFS: 62% 3y DFS: 64% NS
Nagasue et al. [16]	Lap (*n* = 39) Open (*n* = 53)	38 (97%) 53 (100%) *p* = 0.241	NR	NR
Park et al. [17]	Lap (*n* = 71) Open (*n* = 222)	NR	5y OS: 95% 5y OS: 87% *p* = 0.220	5y DFS: 82% 5y DFS: 74% *p* = 0.433
Sammour et al. [18]	Lap (*n* = 89) Open (*n* = 184)	NR	3y OS: 72% 5y OS: 54% 3y OS: 74% 5y OS: 58% *p* = 0.338 *stage II/III only*	3y DFS: 59% 5y DFS: 49% 3y DFS: 65% 5y DFS: 54% *p* = 0.663 *stage II/III only*
Shukla et al. [19]	Lap (*n* = 61) Open (*n* = 22)	61 (100%) 21 (96%) *p* = 0.265	3y OS: 82% 3y OS: 81% *p* = 0.525	3y DFS: 76% 3y DFS: 64% *p* = 0.848
Takahashi et al. [20]	Lap (*n* = 48) Open (*n* = 36)	46 (96%) 35 (97%) *p* = 0.734	3y OS: 94% 3y OS: 80% *p* = 0.441 *stage II/III only*	NR NR *p* = 0.846 *stage II/III only*
Vallribera Valls et al. [21]	Lap (*n* = 69) Open (*n* = 76)	69 (100%) 76 (100%)	3y OS: 72% 5y OS: 62% 3y OS: 75% 5y OS: 62% *p* = 0.715	3y DFS: 60% 5y DFS: 54% 3y DFS: 56% 5y DFS: 49% *p* = 0.688
Vignali et al. [22]	Lap (*n* = 70) t4b (*n* = 18) t4a (*n* = 52) Open (*n* = 70) t4b (*n* = 24) t4a (*n* = 46)	67 (96%) 15 (83%) 52 (100%) 67 (96%) 21 (88%) 46 (100%) *p* = 0.51 *p* = 0.71	5y OS: 53% 5y OS: 42% 5y OS: 59% 5y OS: 59% 5y OS: 48% 5y OS: 64% *p* = 0.18 *p* = 0.42 *p* = 0.71	5y DFS: 47% 5y DFS: 51% *p* = 0.20
Allaix [23]	Lap (*n* = 13)	13 (100%)	3y OS: 69% 5y OS: 54%	3y DFS: 39% 5y DFS: 39%

*DFS* disease-free survival; Lap: laparoscopic surgery; *NR* not reported; *Open* open surgery; *OS* overall survival; *3y* 3-year; *5y* 5-year

* Only study that included M1 patients in survival analyses

**Table 4:** Pooled proportions for laparoscopic and open surgery

Laparoscopic surgery	No. of studies	Sample size	Events	Pooled proportions (95%CI)	*I* ^2^ (%)	*p*-value
Conversion	13	1217	131	0.11 (0.09–0.14)	37	0.175
*Postoperative outcomes*						
Complications	11	669	143	0.23 (0.19–0.27)	24	0.212
Postoperative mortality	8	458	6	0.02 (0.01–0.04)	0	0.841
*Oncological outcomes*						
R0 resection rate	11	1057	927	0.96 (0.91–0.99)	86	<0.001
3y DFS	7	482	302	0.62 (0.57–0.68)	32	0.186
5y DFS	6	400	233	0.58 (0.47–0.68)	75	0.013
3y OS	8	530	407	0.77 (0.71–0.82)	45	0.080
5y OS	6	400	262	0.67 (0.55–0.77)	79	<0.001

Open surgery	No. of studies	Sample size	Events	Pooled proportions (95%CI)	*I* ^2^ (%)	*p*-value
*Postoperative outcomes*						
Complications	10	892	318	0.35 (0.31–0.40)	52	0.029
Postoperative mortality	7	420	9	0.03 (0.02–0.06)	0	0.685
*Oncological outcomes*						
R0 resection rate	10	951	841	0.96 (0.90–0.98)	84	<0.001
3y DFS	6	513	304	0.58 (0.52–0.64)	44	0.115
5y DFS	5	645	389	0.58 (0.47–0.69)	86	<0.001
3y OS	7	549	396	0.72 (0.66–0.78)	52	0.052
5y OS	5	645	445	0.67 (0.53–0.79)	91	<0.001

*DFS* disease-free survival; *OS* overall survival; *3y* 3-year; *5y* 5-year

* The study of Vignali et al left out of pooled survival proportions, due to inclusion of M1 stage

Endpoints used for long-term outcomes varied amongst the studies (Table 3). The pooled proportions are displayed in Table 4. Using meta-analysis, no statistically significant differences were found between laparoscopic and open surgery for any survival measure: RR 1.07 (95% CI: 0.96–1.20) for 3-year DFS, RR 1.04 (95% CI: 0.95–1.15) for 5-year DFS, RR 1.07 (95% CI: 0.99–1.14) for 3-year OS, and RR 1.05 (95% CI: 0.98–1.12) for 5-year OS (Supplementary Table 4).

For subgroup analyses of T4a stage, seven studies were selected of which six provided separate data for pT4a [11, 13, 16, 18, 21, 22] and in one additional study, pT4a was included in >80% in both the open and laparoscopic groups [15]. Pooled proportions of R0 resection of these studies were 0.94 (95% CI: 0.81–0.98) and 0.94 (95% CI: 0.82–0.98) after laparoscopic and open surgery, respectively (RR 1.00, 95% CI: 0.97–1.02, *p* = 0.75 (*I* ^2^ = 0%, *p* = 0.99), 6 studies). Furthermore, no significant difference was found for any survival measure: RR 1.09 (95% CI: 0.93–1.29, 3 studies) for 3-year DFS, RR 1.03 (95% CI: 0.82–1.31, 2 studies) for 5-year DFS, RR 1.11 (95% CI: 1.00–1.25, 3 studies) for 3-year OS, and RR 0.98 (95% CI: 0.83–1.173, 3 studies) for 5-year OS.

Considering T4b stage, five studies provided separate data (Table 3). Pooled proportions of R0 resection of these studies were 0.91 (95% CI: 0.80–0.96) and 0.93 (95% CI: 0.80–0.98) after laparoscopic and open surgery, respectively (RR 0.99, 95% CI: 0.92–1.05, *p* = 0.68 (*I* ^2^ = 0%, *p* = 0.69), 4 studies). Although in favour of open surgery, no significant differences were found for any survival measure: RR 0.91 (95% CI: 0.62–1.34, 1 study) for 3-year DFS, RR 0.91 (95% CI: 0.0.58–1.42, 1 study) for 5-year DFS, RR 0.85 (95% CI: 0.63–1.15, 1 study) for 3-year OS, and RR 0.96 (95% CI: 0.69–1.35, 2 studies) for 5-year OS.

## Discussion

This systematic review of the literature revealed that only observational cohort studies on laparoscopic surgery for T4 colon cancer have been published. The open groups more often included MVRs, and six studies including both pT4a and pT4b stages provided separate outcome data. The pooled proportion of radical resection was 96% for both laparoscopic and open surgery. Analysing evaluable studies for different pT4 stages revealed pooled R0 resection rates for pT4a of 94% in both the laparoscopic and open groups. For pT4b, pooled R0 resection rates were 0.91 and 0.93. Available long-term oncological outcome measures in comparative studies did not show any significant differences between laparoscopic and open surgery for locally advanced colon cancer in both overall, T4a and T4b subgroup analyses.

Given the non-randomized design of the comparative studies, it should be noted that the approach of surgery (laparoscopic or open) has been chosen by the surgeon for specific reasons and with several influencing factors. Criteria on which this decision was made are often hard to determine retrospectively. In general, a laparoscopic approach for T4 colon cancer is used in patients with less extensive tumours requiring less complex procedures. This is confirmed by the comparative studies including MVR, showing a lower proportion of MVR in the laparoscopic groups compared to the open groups. The surgeon’s experience in minimally invasive surgery is crucial, and the laparoscopic and open resections from one institute might have been performed by different surgeons. Furthermore, when there is only peritoneal penetration (T4a), T4 stage is often diagnosed postoperatively, having less influence on the choice of the surgical approach. Achieving an R0 resection of T4a tumours is comparable to T3 tumours, because serosal ingrowth does not threaten margins. T4b tumours invade adjacent structures and require more complex (multivisceral) surgery [26, 27]. Inequality in distribution of T4a and T4b stages between the laparoscopic and open groups may significantly influence the results of comparative studies. In six of the included comparative studies, the proportion of T4b was substantially higher in the open group [14, 15, 17–20]. To overcome these methodological problems, Elnahas et al. excluded T4b stage [13] and Nagasue selected only MVR [24]. Furthermore, three studies applied matching or weighing in order to correct for baseline differences between the two surgical approaches. Only De’Angelis et al. [11] matched for T4a/b stage.

Considering the fact that laparoscopic surgery was likely being performed in more ‘favourable’ T4 colon cancers, one might argue that the pooled 96% radicality of resection should actually be considered worse compared to the similar radicality after open resection of more high-risk T4 cancers. Also the comparable survival probabilities should be interpreted with this allocation bias kept in mind. Selecting (mainly) T4a tumours allows for a more reliable comparison between the surgical approaches. The similar R0 resection rates for laparoscopic and open surgery in this subgroup analysis suggest that laparoscopic surgery for T4a stage is safe. The oncological safety of a minimally invasive multivisceral resection is, however, not clear based on the present data. The pooled radical resection rate after laparoscopic surgery for exclusively T4b stage was only 91%. Allocation bias may particularly play a crucial role in these tumours that invade surrounding organs by probably selecting, for example, a (limited) abdominal wall resection for a laparoscopic approach, whereas extensive multiorgan resections are often performed using open surgery. Future prospective studies differentiating between limited and extensive T4b tumours should point out the suitability of laparoscopic surgery for (part of) the T4b subgroup.

The risk of developing a postoperative complication was significantly lower after laparoscopic surgery as compared to open surgery (RR 0.65; 95% CI: 0.55–0.77, *p* < 0.001). However, in five out of ten comparative studies reporting on postoperative complications, substantially more MVRs were performed in the open group as compared to the laparoscopic group (Supplementary Table 3) [14, 15, 17, 19, 22]. A MVR is accompanied by more postoperative complications than just a segmental colectomy [26, 27]. The benefit of a laparoscopic approach for MVR might be limited, especially considering the fact that often large extraction incisions have to be made to remove the relatively large MVR specimens. Furthermore, in the open subgroups of three studies [15, 18, 19], significantly higher proportions of emergency procedures were included, with its impact on postoperative complications [28].

The influence of expertise on oncological outcomes is suggested based on a wide range in reported R0 resection rates of 74–100%. The study of Elnahas et al. [13] was a multi-institutional cohort study and used data from a (bi)nationwide database, showing overall R0 resection rates of 75%. Information on surgical experience and hospital volume was not provided, but the nationwide character of the study suggests a substantial contribution of non-expert centres. Potentially, centralization of care contributes to better R0 resection rates.

This systematic review has several limitations. An important limitation is the relatively low quality of evidence of the included studies as already discussed. Only three multicentre studies were included, suggesting publication bias of single-centre studies originating from expert centres. Furthermore, substantial heterogeneity between the studies exists because of the different patient populations assessed with regard to definition of T4 (clinical T4 versus pathological T4, T4a versus T4b). Duration of follow-up was relatively short, with two studies reporting on 3-year oncological outcomes [15, 29], while median follow-up was only 27 and 32 months, respectively. Also, with conversion rates of up to 23%, the intention to treat analysis might muddle the reported outcomes.

In conclusion, current literature does not provide the definitive answer on oncological safety of minimally invasive surgery for locally advanced colon cancer. Based on subgroup analysis, we cautiously conclude that laparoscopic surgery for T4a tumours might be safe, whereas it seems less appropriate for T4b tumours requiring multivisceral resection. Laparoscopic resection of T4 colon cancer should probably only be performed in selected patients by experienced surgeons. These selection criteria and level of experience have to be defined more precisely in future studies.

## Electronic supplementary material

Below is the link to the electronic supplementary material.

Supplementary material 1 (DOC 88 kb)

Supplementary material 2 (JPEG 77 kb)

Supplementary material 3 (DOC 33 kb)

Supplementary material 4 (DOC 42 kb)

Supplementary material 5 (DOC 45 kb)

Supplementary material 6 (DOC 35 kb)
