# Supplementary figures and images for: Laparoscopic surgery for T4 colon cancer: a systematic review and meta-analysis
Source: Surg Endosc. 2017 Apr 21;31(12):4902–12. doi: 10.1007/s00464-017-5544-7 (PMC5715041; doi:10.1007/s00464-017-5544-7)

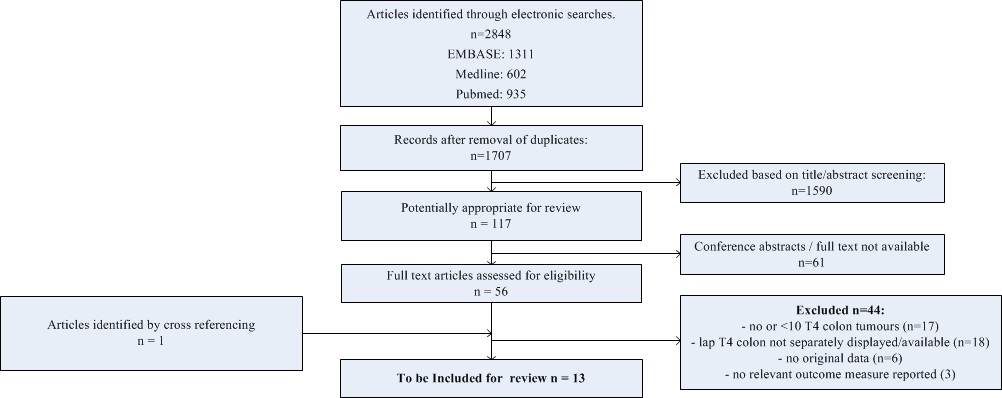


*Suppl. figure 1. Search results and selection of included studies.*

Supplement: Supplementary file 1 — Supplementary material 1 (DOC 88 kb) [file 464_2017_5544_MOESM1_ESM.doc]

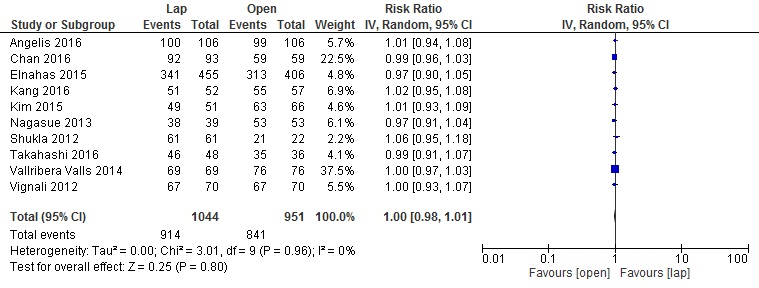

Supplement: Supplementary file 2 — Supplementary material 2 (JPEG 77 kb) [file 464_2017_5544_MOESM2_ESM.jpg]
