# Supplementary material for: Laparoscopic surgery for T4 colon cancer: a systematic review and meta-analysis
Source: Surg Endosc. 2017 Apr 21;31(12):4902–12. doi: 10.1007/s00464-017-5544-7 (PMC5715041; doi:10.1007/s00464-017-5544-7)
Supplement: Supplementary file 3 — Supplementary material 3 (DOC 33 kb) [file 464_2017_5544_MOESM3_ESM.doc]

| **Embase**  hits: 1311 | (exp colon carcinoma/ or colorectal cancer/ or colon cancer/ or (colorectal neoplasms or colorectal neoplasm* or colorectal malignan* or colorectal tumor or colorectal tumour* or colorectal carcinoma or colonic neoplasms or colonic neoplasm* or colon neoplasm* or colon cancer* or colonic cancer* or colonic malignan* or colon tumor* or colon tumour*).ti, ab, kw.) |
| --- | --- |
|  | AND ((locally advanced* or T4 or pT4 or cT4 multivisceral* or advanced*).ti, ab, kw.) |
|  | AND (exp laparoscopic surgery/ or (laparoscopy or laparoscop* or open* or minimally invasive* or closed* or minimal invasive*).ti, ab, kw.) |
| **Medline via Ovid**  hits: 602 | *Colorectal Neoplasms/di, th or *Colonic Neoplasms/di, th or (colorectal neoplasms or colorectal neoplasm* or colorectal malignan* or colorectal tumor or colorectal tumour* or colorectal cancer or colorectal carcinoma or colonic neoplasms or colonic neoplasm* or colon neoplasm* or colon cancer* or colonic cancer* or colonic malignan* or colon tumor or colon tumour or colon carcinoma).ti, ab, kw. |
|  | AND ((locally advanced* or T4 or pT4 or cT4 or multivisceral* or advanced*).ti, ab, kw.) |
|  | AND (*Laparoscopy/di, th or (laparoscopy or laparoscop* or open* or minimally invasive* or closed* or minimal invasive*).ti, ab, kw.) |
| **Pubmed via Medline**  hits: 935 | ("Colorectal Neoplasms"[Mesh] OR Colorectal neoplasm*[tiab] OR Colorectal cancer*[tiab] OR Colorectal cancer*[tiab] OR Colorectal malignan*[tiab] OR Colorectal tumor*[tiab] OR Colorectal tumour*[tiab] OR Colorectal carcinoma*[tiab] OR "Colonic Neoplasms"[Mesh] OR Colonic Neoplasm*[tiab] OR Colon Neoplasm*[tiab] OR Colon Cancer*[tiab] OR Colonic Cancer*[tiab] OR Colonic malignan*[tiab] OR Colon tumor*[tiab] OR Colon tumour*[tiab] OR colon carcinoma*[tiab]) |
|  | AND (Locally advanced *[tiab] OR T4[tiab] OR multivisceral*[tiab] OR advanced*[tiab] OR pT4[tiab] OR cT4[tiab]) |
|  | AND ("Laparoscopy"[Mesh] OR laparoscop*[tiab] OR open*[tiab] OR minimally invasive*[tiab] OR closed*[tiab] OR minimal invasive*[tiab]) |

*Suppl. table 1. Search strategy.* Performed on February 21^st^ 2017
