# Supplementary material for: Laparoscopic surgery for T4 colon cancer: a systematic review and meta-analysis
Source: Surg Endosc. 2017 Apr 21;31(12):4902–12. doi: 10.1007/s00464-017-5544-7 (PMC5715041; doi:10.1007/s00464-017-5544-7)
Supplement: Supplementary file 4 — Supplementary material 4 (DOC 42 kb) [file 464_2017_5544_MOESM4_ESM.doc]

|  | Selection | | | | Comparability | Outcome | | | Total |
| --- | --- | --- | --- | --- | --- | --- | --- | --- | --- |
|  | Representativeness of the exposed cohort | Selection of the non-exposed cohort | Ascertainment of exposure | Outcome of Interest | Comparability of the cohorts on baseline | Assess-ment of the outcome | Follow-up long enough | Adequacy follow-up |  |
| **De'Angelis et al.** | * | * | * | * | ** | * | * | - | 8 |
| **Chan et al.** | * | * | * | * |  | * | * |  | 6 |
| **Elnahas et al.** | * | * | * | * | ** | * | * | * | 9 |
| **Kang et al** | * | * | * | * | - | * | * | * | 7 |
| **Kim et al.** | * | * | * | * | - | * | * | * | 7 |
| **Nagasue et al.** | - | * | * | * | - | * | * | - | 5 |
| **Park et al.** | * | * | * | * | - | * | - | - | 5 |
| **Sammour et al.** | * | * | * | * | - | * | * | * | 7 |
| **Shukla et al.** | * | * | * | * | - | * | * | - | 6 |
| **Takahashi et al.** | * | * | * | * | - | * | * | - | 6 |
| **Vallribera Valls et al.** | * | * | * | * | - | * | * | - | 6 |
| **Vignali et al.** | * | * | * | * | ** | * | * | * | 9 |
| **Allaix et al.** | * | - | * | * | - | * | * | * | 6 |

*Suppl. table 2.Quality of the included studies using the Newcastle-Ottawa Scale.*
