# Supplementary material for: Laparoscopic surgery for T4 colon cancer: a systematic review and meta-analysis
Source: Surg Endosc. 2017 Apr 21;31(12):4902–12. doi: 10.1007/s00464-017-5544-7 (PMC5715041; doi:10.1007/s00464-017-5544-7)
Supplement: Supplementary file 5 — Supplementary material 5 (DOC 45 kb) [file 464_2017_5544_MOESM5_ESM.doc]

| **Author, year** | **Group** | **Conversion** | **Years of surgery, volume** | **Emergency procedures** | **Multivisceral resections** | **Postoperative complications** | **Postoperative mortality** |
| --- | --- | --- | --- | --- | --- | --- | --- |
| **De’Angelis et al, 2016** | Lap (n=106)  Open (n=106) | 13 (12%) | 2005-2014  11 | 0 (0%)  0 (0%) | 15 (14%)  20 (19%)  p=0.460 | 30 (29%)  36 (35%)  p=0.372 | 1 (0.9%)  4 (3.8%)  p=0.369 |
| **Chan et al, 2016** | Lap (n=93)  Open (n=59) | 8 (8.7%) | 2008-2014  13 | 0 (0%)  0 (0%) | - | NR | NR |
| **Elnahas et al, 2015** | Lap (n=455)  Open (n=406) | 48 (11%) | 2011 -2012  NA | 83 (19%)  80 (20%)  p=0.70 | - | NR | NR |
| **Kang et al, 2016** | Lap (n=52)  Open (n=57) | 4 (7.7%) | 3/2003- 6/2013  5 | 1 (2%)  2 (3.5%)  p=1.0 | 7 (14%)  21 (37%)  p=0.005 | 7 (14%)  21 (37%)  p=0.005 | 1 (1.9%)  0 (0%)  p=0.477 |
| **Kim et al, 2015** | Lap (n=51)  Open (n=66) | 7 (14%) | 2008-2013  8.5 | 1 (2.1%)  8 (24%)  p=0.002* | 14 (28%)  35 (53%)  p=0.005* | 12 (24%)  24 (36%) | NR  NR |
| **Nagasue et al, 2013** | Lap (n=39)  Open (n=53) | 3 (7.7%) | 2005-2012  4.9 | 0 (0%)  0 (0%) | 39 (100%)  53 (100%) | 8 (21%)  11 (21%)  p=0.977 | 0 (0%)  0 (0%) |
| **Park et al, 2016** | Lap (n=71)  Open (n=222) | 4 (5.6%) | 2000-2010  6.5 | NR | 7 (9.9%)  73 (33%)  p<0.001 | 10 (14%)  70 (32%)  p=0.004 | NR |
| **Sammour et al, 2015** | Lap (n=89)  Open (n=184) | 13 (15%) | 2003-2009  NA | 11 (12%)  82 (45%)  p=0.000 | NR | 26 (30%)  79 (44%)  p=0.030 | NR |
| **Shukla et al, 2015** | Lap (n=61)  Open (n=22) | 13 (21%) | 2003-2011  6.8 | 2 (3%)  5 (23%)  p=0.013* | 14 (23%)  9 (41%)  p=0.107 | 17 (28%)  8 (36%)  0.467 | 0 (0%)  0 (0%) |
| **Takahashi et al, 2017** | Lap (n=48)  Open (n=36) | 6 (13%) | 6/2005-2014  5.1 | NR | 48 (100%)  36 (100%) | 8 (17%)  13 (36%)  p=0.042 | 0 (0%)  0 (0%) |
| **Vallribera Valls, 2014** | Lap (n=69)  Open (n=76) | 4 (5.9%) | 2005 – 5/2009  16 | 0 (0%) | - | 18 (26%)  37 (49%)  p=0.005 | 3 (4.3%)  4 (5.3%)  p=0.797 |
| **Vignali et al, 2012** | Lap (n=70)  Open (n=70) | 5 (7.1%) | 2002-2012  6.4 | 0 (0%)  0 (0%) | 18 (26%)  24 (34%) | 15 (21%)  19 (28%)  p=0.42 | 1 (1.4%)  1 (1.4%) |
| **Allaix et al, 2013** | Lap (n=13) | 3 (23%) | 1993-2007  1 | 0 (0%) | NR | 1 (7.7%) | 0 (0%) |

*Suppl. table 3. Operative and postoperative details.* Lap: laparoscopic surgery; NR: not reported; Open: open surgery; Years of surgery: years the surgery was performed; Volume: number of resections performed per hospital annually.
