# Supplementary material for: Laparoscopic surgery for T4 colon cancer: a systematic review and meta-analysis
Source: Surg Endosc. 2017 Apr 21;31(12):4902–12. doi: 10.1007/s00464-017-5544-7 (PMC5715041; doi:10.1007/s00464-017-5544-7)
Supplement: Supplementary file 6 — Supplementary material 6 (DOC 35 kb) [file 464_2017_5544_MOESM6_ESM.doc]

|  |  | **Laparoscopy** | | **Open** |  |  |  | **Heterogeneity** | |
| --- | --- | --- | --- | --- | --- | --- | --- | --- | --- |
| **Oncological outcomes** | No. Of studies | Sample size | Events* | Sample size | Events* | Risk Ratio (95%CI) | *p*-value | I^2^ | *p*-value |
| R0 resections | 10 | 1044 | 914 | 951 | 841 | 1.00 (0.98-1.01) | 0.80 | 0% | 0.96 |
| 3 year DFS | 6 | 469 | 297 | 513 | 304 | 1.07 (0.96-1.20) | 0.23 | 9% | 0.36 |
| 5 year DFS | 6 | 457 | 261 | 715 | 425 | 1.04 (0.95-1.15) | 0.36 | 0% | 0.48 |
| 3 year OS | 7 | 517 | 398 | 549 | 396 | 1.07 (0.99-1.14) | 0.08 | 0% | 0.46 |
| 5 year OS | 6 | 457 | 290 | 715 | 486 | 1.05 (0.98-1.12) | 0.16 | 0% | 0.67 |

*Suppl. table 4. Meta-analyses for oncological outcomes comparing laparoscopic versus open surgery for T4 colon cancer.* DF: disease free; DFS: disease free survival; OS: overall survival; 3y: 3 year; 5y: 5 year. *Events: number of R0 resections / number of patients (disease free) alive.
